# Effectiveness of a diabetes program based on digital health on capacity building and quality of care in type 2 diabetes: a pragmatic quasi-experimental study

**DOI:** 10.1186/s12913-023-09082-7

**Published:** 2023-01-31

**Authors:** Daniela Moraes Morelli, Fernando Rubinstein, Marilina Santero, Luz Gibbons, Daniela Moyano, Analia Nejamis, Andrea Beratarrechea

**Affiliations:** Instituto de Efectividad Clínica y Sanitaria, Buenos Aires, Argentina

**Keywords:** Diabetes, Digital health, Primary care, Patient education

## Abstract

**Abstract:**

Health systems in Latin America face many challenges in controlling the increasing burden of diabetes. Digital health interventions are a promise for the provision of care, especially in developing countries where mobile technology has a high penetration. This study evaluated the effectiveness of the implementation of a Diabetes Program (DP) that included digital health interventions to improve the quality of care of persons with type 2 Diabetes (T2DM) in a vulnerable population attending the public primary care network.

**Materials and methods:**

A quasi-experimental pre-post uncontrolled study was conducted in 19 primary care centers and hospitals in the province of Corrientes, Argentina. We included persons with T2DM, age >  = 18 years with access to a mobile phone. The multicomponent intervention included a mobile app with a diabetes registry, a clinical decision support tool for providers and a text messaging intervention for patients.

**Results and discussion:**

One thousand sixty-five participants were included, 72.8% had less than 12 years of formal education and 53.5% lacked health coverage. Comorbidities were hypertension (60.8%) and overweight/obesity (88.2%). During follow-up there was a significant increase in the proportion of participants who underwent laboratory check-ups (HbA1c 20.3%-64.4%; *p* < 0.01) and foot exams (62.1%-87.2%; *p* < 0.01). No changes were observed at 12 and 24 months in the proportion of participants with poor metabolic control. The proportion of participants with uncontrolled blood pressure (≥ 140/90 mmHg) decreased from 47.2% at baseline to 30.8% at 24 months in those with a follow-up visit.

**Conclusion:**

The DP was innovative by integrating digital health interventions in the public primary care level. The study showed improvements in quality indicators related with diabetes care processes and in blood pressure control.

## Introduction

Global statistics show that currently 1 in 11 people lives with diabetes, corresponding to 9.3% of the adults aged 20 to 79 years. Variability is observed in the prevalence and in the capacity of the countries to prevent and control diabetes, particularly in low-and-middle-income countries (LMIC). [1–3]

Estimates from the International Diabetes Federation (IDF) indicate that in Latin America (LA) in 2019 there were 243.200 deaths from diabetes in people between 20 and 79 years and $ 67.7 billion accumulated in health expenses [4]. Likewise, projections suggest an increase in these numbers in the LMIC, identified as one of the main causes of premature death, disability and morbidity [5, 6]. In this sense, Argentina showed a significant increase in the prevalence of diabetes from 8.4% to 12.7% between 2009–2018, accompanied by a rise in the prevalence of obesity and low physical activity, both recognized risk factors for type 2 diabetes mellitus (T2DM) [7].

Health systems in LA face many challenges in controlling the increasing burden of diabetes. Although most LA countries have made adjustments to incorporate evidence-based interventions to address chronic diseases and improve quality of care, these interventions have not been widely implemented and adopted in clinical practice and results are far from optimal in the region [8–11].

Given the increasing prevalence of diabetes there is a need for innovative and effective ways to deliver interventions to improve diabetes care in health systems with limited resources. Moreover, evidence indicates that digital health interventions are a promise for the provision of care, especially in developing countries where mobile technology has a high penetration. A systematic review evaluating mHealth interventions in Non-Communicable Diseases (NCDs) from 20 randomized controlled trials from 14 LMIC countries found that one-way SMS was the most widely used mobile function for sending reminders, health education, and information. [12] The recommendations for Blood Pressure Measurement in Humans and Experimental Animals mentions that devices capable of transmitting readings by telephone have the potential to improve patient compliance and therefore blood pressure control. [13] A meta-analysis that evaluated 39 studies on the effectiveness of social media (*n* = 4) and mobile health (*n* = 35) concluded that cancer screening programs should consider mHealth interventions due to their promising role in advocacy of participating in cancer screenings. [14]

Although the use of digital health has been gaining prominence to strengthen health care systems in the control of chronic diseases, caution is required to support its routine use in LMIC and dictate policies for its adoption [15–20]. Success of digital interventions depends not only on the digital solution but on how the system is implemented and integrated into the healthcare system. Aligned with this need and context, this study evaluated the implementation of a comprehensive diabetes program (PD) in the province of Corrientes, Argentina, that included digital health interventions and capacity building [21, 22] to improve the quality of care of persons with type 2 diabetes attending the public primary care level.

## Materials and methods

### Study design

Quasi-experimental pre-post uncontrolled study in 19 public primary care clinics (PCCs) and hospitals in the province of Corrientes, Argentina. Details of the study design have already been published [22].

### Setting and participants

We included primary care services of hospitals and clinics from the public primary care network of the Province of Corrientes. Corrientes is located in the northeast of Argentina, has more than one million inhabitants, 41.2% are below the poverty line and 48% do not have health insurance. [23–25]. To be included, sites had to be located in poor urban areas, have at least 800 outpatient visits each month; employed community health workers, be affiliated to the REMEDIAR and REDES Program (provides free chronic medications) and have an internet connection. Each site recruited between 50 and 100 participants.

Participants with a confirmed diagnosis of T2DM, age >  = 18 years, with access to mobile phones, living less than 10 km from the site and who voluntarily signed the informed consent were included. Pregnant women at the time of screening and bedridden people were excluded.

### Intervention

**Fig. 1:**
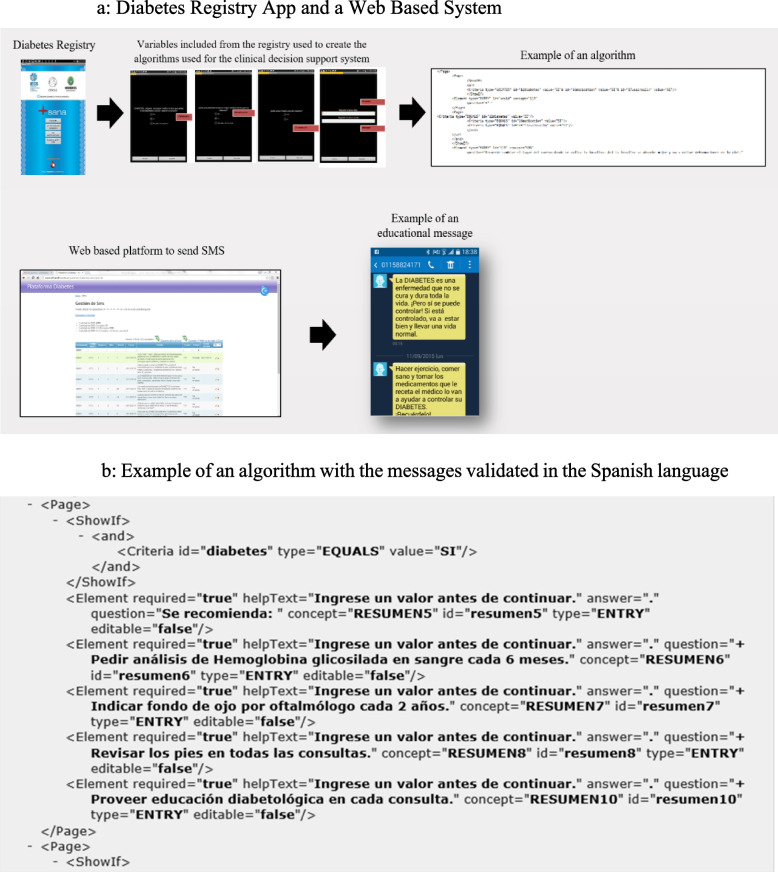
**a** Diabetes registry app and a web based system. **b**. Example of an algorithm with the messages validated in the Spanish language

**Fig. 2:**
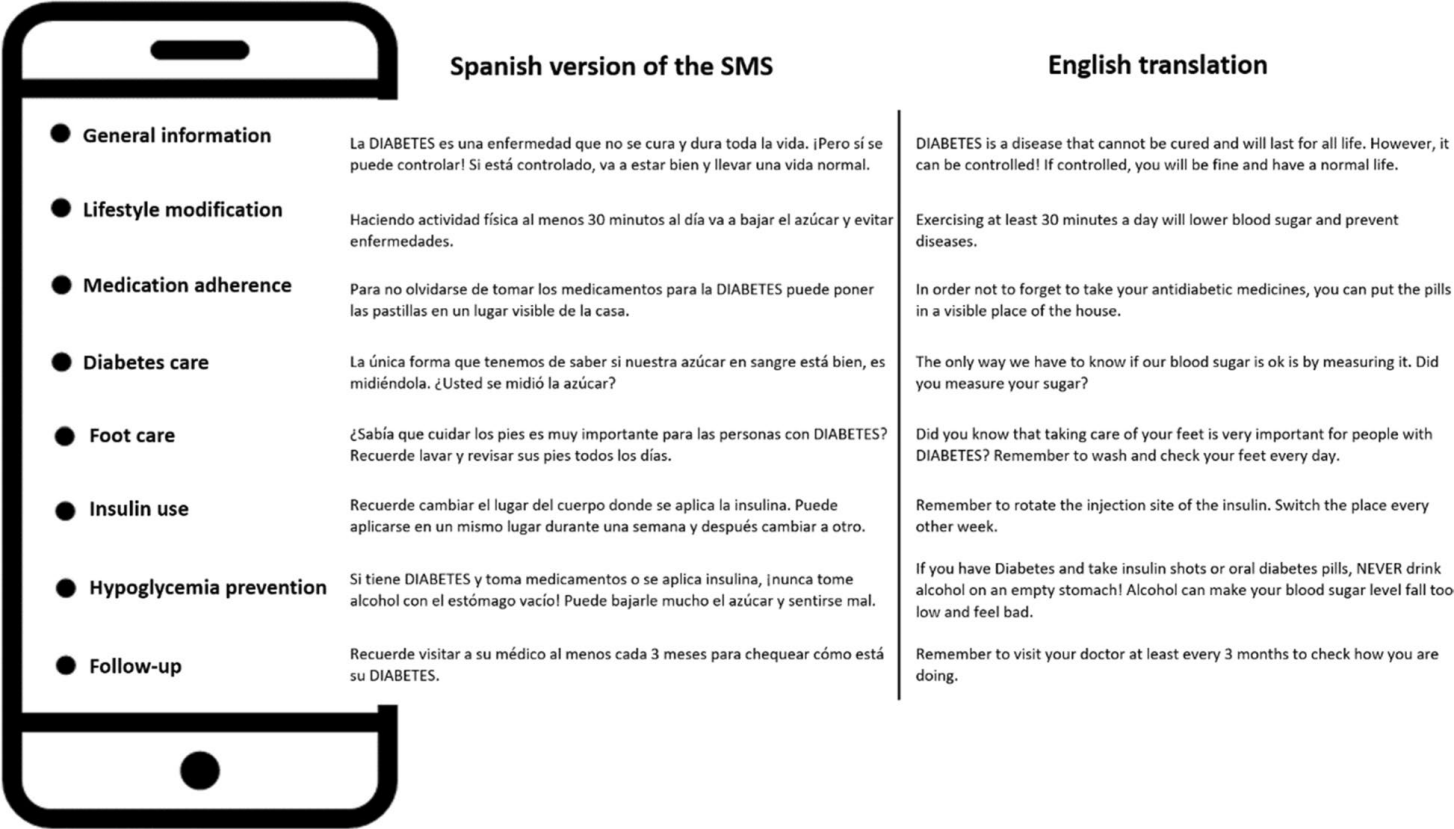
Examples of validated short text messages (SMS)

The Diabetes Program includes a multi-component intervention to improve diabetes management and control at a system, provider and patient level:

- 1. *Primary care team training.* We held on-site two-day intensive workshops for primary care physicians, nurses and community health workers (CHWs) focused on diagnosis, diabetes stepped-care management, self-management education, chronic follow-up and diabetes prevention.
- 2. *Educational outreach visits.* Educational outreach visits were conducted to audit, address barriers to the implementation of clinical practice guidelines (CPG) and provide feedback to health care professionals about their performance.
- 3. *Digital Health strategies.* We developed an information and communication system that included a diabetes registry app and a web-based platform (Figure 1a and b) to deliver educational text messages to the participants. The system collects data during the clinical encounters, provides a decision support tool based on CPG to guide diabetes care, monitors the persons with diabetes and provides diabetes education through previously validated bespoke SMS text messages (Figure 2) [23].

### Data collection

At each visit, the health provider collected data from the participants in accordance with the study protocol and CPG, in the Diabetes Registry, which was installed on a tablet. Process and intermediate results indicators related with timely screening for complications and comorbidities, pharmacological treatment and health outcomes were used to evaluate the quality of care.

### Data analysis

Descriptive data on demographic and baseline clinical characteristics of the sample are reported as median and interquartile range (IQR) for continuous variables and percentages for categorical variables. We built a dynamic cohort; and estimated the number of participants who had at least one follow up visit within the first year (as recommended by CPG) and for those who had at least a visit from 12 to 24 months. Process and intermediate health outcome indicators for the study population were estimated for baseline, 12 and 24 months of follow up. In addition, some indicators such as level of control of HbA1c and blood pressure (BP) were reported by visit for those who had a registry on that visit. We used chi2 test for trends to evaluate if changes were significant along the study period. Finally, we estimated the initial measurement of HbA1c, BP and defined poor initial control based on those baseline measurements according to CPG [24]. For those participants who had more than one visit we defined the last measurement as the final level of control.

### Ethics approval and consent to participate

This study was approved by the Institutional Review Board “Hospital Italiano de Buenos Comité de Ética de Protocolos de Investigación (CEPI)”, No. 2641, 22/10/2015. All the participants signed an informed consent, and the confidentiality of the information was guaranteed. All methods were carried out in accordance with relevant guidelines and regulations.

## Results and discussion

**Fig. 3:**
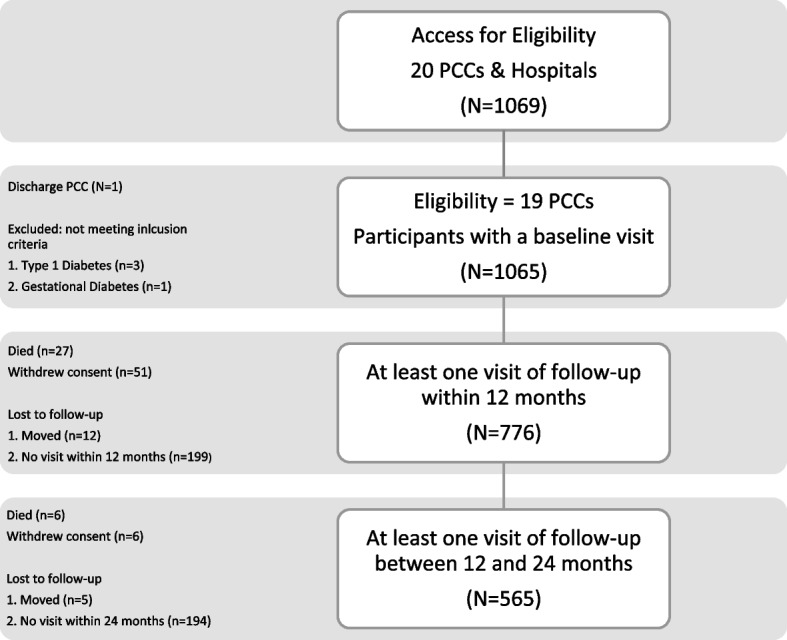
Diabetes program enrollment and follow-up flowchart

Nineteen primary care clinics (PCCs) and hospitals, 36.7% in the City of Corrientes and the rest distributed in other departments of the Province. 1065 participants were enrolled between November 2015 and February 2017, and followed until May 2018 (Fig. 3).

The majority were older than 45 years, mainly women, with low level of education and half had no health coverage. Common comorbidities were hypertension (60.8%) and overweight/obesity (88.2%); 178 (16.7%) were not receiving treatment for T2DM upon entering the study. Among those who received treatment, metformin was the drug most frequently used (726/845; 85.9%), exclusively or in combination with another antidiabetic drug (Table 1). The median follow-up of the participants in our cohort was 527 days (IQR 358–716), and 27.1% only had the baseline visit throughout the study.

**Table 1:** Characteristics of study participants at baseline (*N* = 1065)

Characteristic	n (%)	Me (IQR)
**Socio-demographic**		
Age in years	N/A	54 (47 – 61)
Women	657 (61.7%)	N/A
Married or living with a partner	575 (54%)	N/A
Less than 12 years of formal education	775 (72.8%)	N/A
No health insurance	570 (53.5%)	N/A
Mean age at diabetes diagnosis	N/A	48 (40 – 55)
Treatment for T2DM	887 (83.3%)	N/A
**Medications**		
Only oral antidiabetics drugs	750 (70.4%)	N/A
Only Insulin	42 (3.9%)	N/A
Insulin + oral antidiabetic drug	95 (8.9%)	N/A
Calcium channel blocker (amlodipine)	88 (8.3%)	N/A
Beta blocker (atenolol)	54 (5.1%)	N/A
ACE inhibitor (enalapril)	446 (41.9%)	N/A
ACE Inhibitor ARAII (losartan)	109 (10.2%)	N/A
Diuretics (thiazides or furosemide)	80 (7.5%)	N/A
Statins (atorvastatin or simvastatin)	179 (16.8%)	N/A
Aspirin	172 (16.1%)	N/A
**Lifestyle**		
Smoking^#^	120 (11.3%)	N/A
Alcohol consumption^#^	203 (19.1%)	N/A
Physical activity^#^	338 (31.7%)	N/A
Five or more fruits or vegetables per day^#^	326 (30.6%)	N/A
**Comorbidities**		
Hypertension^#^	648 (60.8%)	N/A
Dyslipidemia^#^	350 (32.9%)	N/A
Overweight /Obesity (BMI ≥ 25)	939 (88.2%)	N/A
Cardiovascular risk^*^		
Low	369 (34.6%)	N/A
Moderate	200 (18.8%)	N/A
High	162 (15.2%)	N/A
Very high	334 (31%)	N/A
**Acute complications**		
At least one episode of severe Hypoglycemia ^#^	89 (8.4%)	N/A
**Macrovascular chronic complications**^**#**^		
Stroke	38 (3.6%)	N/A
Angioplasty, bypass or stent	19 (1.8%)	N/A
Peripheral arteriopathy	40 (3.8%)	N/A
Myocardial infarction	32 (3%)	N/A
**Microvascular chronic complications**^**#**^		
Retinopathy, photocoagulation or blindness	111 (10.4%)	N/A
Chronic kidney disease	24 (2.2%)	N/A
Peripheral neuropathy or amputation	33 (3.1%)	N/A
**Clinical and metabolic profile**		
SBP (mmHg)	1055 (99.1%)	130 (120–146.5)
DBP (mmHg)	1055 (99.1%)	80 (70–90)
HbA1c (%)	216 (20.3%)	8 (6.9–9.9)
Fasting blood glucose (mg/dL)	509 (47.8%)	168.59 (128–235)
Hemoglucotest	180 (16.9%)	184.5 (136- 180)
Total cholesterol (mg/dL)	387 (36.3%)	194 (160–227.7)
LDL (mg/dL)	186 (17.5%)	108.50 (79–135)
HDL (mg/dL)	184 (17.3%)	42.50 (35.5–53.5)
Triglycerides (mg/dL)	332 (31.2%)	158 (115–231)
Microalbuminuria (mg/dL)	33 (3.1%)	11.3 (4.3–100)
Creatinine (mg/dL)	102 (9.6%)	1 (1–1)

^#^self-reported

^*^2007 WHO/ISH cardiovascular risk prediction charts for the Americas AMR B; N/A = not applicable; Me = median; IQR = interquartile range

### Implementation indicators

#### Primary care team training

We trained 59 physicians, 193 nurses, 231 CHWs to implement CPG and 231 health care professionals participated in workshops to provide self-management education. Forty self-management workshops were implemented in selected PCCs and hospitals and 1,118 persons with diabetes participated in group self-care workshops. We also trained 54 non- professionals peers, who had diabetes or close familiarity with its management. Educational materials (i.e. diabetes CPG, manual for diabetes education, manual for diabetes self-care and educational kits for patients) were provided to both health professionals and persons with T2DM.

Through an open source educational platform, 2 self-administered virtual modality courses were implemented for the management of persons with T2DM at the primary care level, one for physicians and nurses and the other for CHWs. Online learning was configured as a knowledge update strategy to take advantage of time, due to the flexibility it posed. [25] Health care professionals who participated in the workshops were motivated to participate in the online courses. However, among those who registered in the online educational platform, we found a low participation rate at 12 months since only 47.3% of physicians and nurses (96 of 203) and 62.5% of CHWs (70 of 112) completed the course.

#### Digital health strategies

Forty-four health care professionals were trained to use the diabetes registry installed on the tablets and 21 (47.7%) continued using the registry at the end of the study. The diabetes registry included a clinical decision support system to guide the implementation of CPG among providers.

Regarding the text messaging, we developed and validated 113 messages that were grouped into different subsets (Table 2). We used formative research from a previous study to develop these messages [26]. Specific algorithms that used data from the diabetes registry retrieved a personalized set of SMS messages according to the risk profile of each patient. (Supplemental material). We used SMS Gateway program to send the messages [27]. The majority of the enrolled participants (81.6%) had access to a cell phone and 809 accepted to receive weekly educational messages. A total of 43331 SMS were sent with a median of 57 (range 1–122) per participant. Because of the customization, the number of SMS according to each category varied (Table 2).

**Table 2:** Messages sent by domain

Domains of educational messages	Number of SMS sent
General information	12,963
Lifestyle modification	11,350
Medication adherence	5805
Diabetes care	3397
Foot care	3623
Insulin use	1802
Hypoglycemia prevention	1386
Follow-up	3005
Undelivered SMS ^#^	1581

^#^phone line was not active at the time of delivery of the SMS

#### Educational outreach visits

We performed two outreach visits to each PCCs and hospitals where we reviewed processes related with diabetes care with the primary care teams. In addition, we shared the indicator results obtained with local healthcare authorities and discussed which were the barriers and challenges to improve population health management.

### Process and intermediate health outcome indicators

#### Baseline

We found that persons with T2DM had low access to lab and to specialist care. Although the majority were under antidiabetic treatment; half of those with HbA1c measurements had poor metabolic control (HbA1c levels ≥ 8%).

Of the participants who reported hypertension, 86.6% received hypertensive medication but 47.2% were poorly controlled. Only 17.8% between 40 and 75 years of age were under statin treatment (Table 3).

**Table 3:** Indicators of quality of care for persons with T2DM –processes and intermediate results

**Indicators**	**Baseline Visit (*N* = 1065)**	**Follow-up visit within 12 months**^**#**^** (*N* = 776)**	**Follow-up visit** **12—24 months**^**#**^** (*N* = 565)**	p^*^
	**n (%)**	**n (%)**	**n (%)**	
**Process indicators**				
HbA1c testing	216 (20.3%)	268 (34.5%)	364 (64.4%)	< 0.01
Cholesterol LDL testing	387 (36.3%)	229 (29.5%)	407 (72%)	< 0.01
Triglycerides testing	332 (31.2%)	205 (26.4%)	363 (64.2%)	< 0.01
Creatinine testing	102 (9.6%)	365 (34.3%)	496 (46.6%)	< 0.01
Blood pressure measurement	1055 (99.1%)	776 (100%)	538 (95.2%)	NS
Monofilament—foot exam performed	662 (62.1%) 230 (21.6%)	666 (85.8%) 278 (35.8%)	493 (87.2%) 186 (32.9%)	< 0.01 < 0.01
Eye exam performed	887 (83.3%)	722 (93%)	553 (97.9%)	< 0.01
Any treatment for diabetes^$^	561/648	529/617	407/464	NS
Hypertensive^#^ on antihypertensive medication	(86.6%) 162/911	(85.7%) 194/691	(87.7%) 158/507	< 0.01
Age > = 40 & < = 75 years on statins	(17.8%)	(28.1%)	(31.2%)	
**Intermediate Results**				
Poor metabolic control (HbA1c > = 8%)^#^	109/216 (50.5%)	121/268 (45.1%)	177/364 (48.6%)	NS < 0.01
Uncontrolled blood pressure (> = 140/90 mmHg) ^#^	499/1055 (47.2%)	325/776 (41.9%)	166/538 (30.8%)	

^*^p significant < 0.05, *NS* non-significant, *N/A* not applicable; chi2 test for trends

^#^ based on those with a follow-up visit

^$^oral antidiabetics drugs, insulin or both

#### Follow-up

Regarding follow up visits, 72.8% complied with a visit within 12 months and 53% between 12 and 24 months. During the follow-up period there was a significant increase in the proportion of persons with T2DM who underwent laboratory check-ups and foot exams. Antidiabetic and statin treatment also increased in this period. But, although the proportion of persons with T2DM who had accessed to antidiabetic treatment and to HbA1c lab increased, it did not result in better metabolic control.

Lastly, among people with hypertension, most had pharmacological treatment which was continued throughout the follow-up period and led to a 16% improvement in blood pressure control.

In low resources scenarios, diabetes guidelines recommend at least one control visit and one HbA1c testing within one year after the initial evaluation (ideally one visit every 6 months), yearly foot exam and an eye exam every one to two years. Since participants had different follow up times through the study period (from up to six months to full 24 months), those who had at least one visit every 6 to 8-month during the study, one HbA1C test in the previous 12 months and a foot exam performed were considered adherent to the clinical practice guideline recommendations.

Table 4 shows the cohort stratified by the different follow up times and the proportion within recommended guidelines in each group (i.e., out of the 247 patients who were followed between 12 and 18 months, 162 (65.6%)) complied with the definition [21].

**Table 4:** Proportion of patients who complied within CPG recommendations by follow up time

**Follow-up time**	Up to 6 months^*^	**6 – 12 months**	**12 – 18 months**	**18 – 24 months**	**Total**
N patients	347	145	247	326	1065
Comply with CPG, n (%)	72 (20.7)	88 (60.7)	162 (65.6)	183 (56.1)	505 (47.4)

^*^Patients with only one visit (baseline) were considered outside the CGP recommendations

## Discussion

The majority of the participants were middle aged women, had low educational level and relied exclusively on public health coverage. The sociodemographic profile of our cohort was similar to other studies conducted in healthcare centers located in low resource settings in Latin America [28, 29]. Although persons with T2DM had a low educational attainment, the majority had access to a cell phone and agreed to receive SMS educational messages as part of the intervention. Recently, a cross-sectional study conducted in Ethiopia (n = 423) showed a high access to cell phones, 77.8% of the patients with diabetes had access to a cell phone and 70.5% were willing to receive health messages on their mobile [13].

Argentina has a high number of mobile cellular subscriptions per inhabitant (125.8 per 100 people) with a smartphone adoption in 2019 of 65% [30]. The majority had access to cellphones so this was not a barrier for the inclusion and follow-up of people with diabetes in the study [31, 32].

Components of the DP were implemented in a pragmatic way, within the limitations generated by the local context and an overburdened provincial public health system. In this sense, during the implementation, many PCCs were forced to evacuate because of river floods. And after the floods, Corrientes was affected by a dengue fever epidemic affecting the implementation of the DP during the outbreak.

Compliance to face to face training activities was higher than to remote learning activities among healthcare professionals. Barriers related with lack of time, infrastructure and technology skills (e.g. not having access to a personal computer or an email) as well as poor internet connectivity might be related with not engaging or withdrawals from on-line courses as reported in other studies [33, 34]. In the prepandemic there wasn´t an institutional strategy in the province to support the use of online tools for capacity building.

The results of the analysis of the quality indicators suggest that the synergy of multiple strategies to strengthen the primary care level promoted favorable changes in the management of T2DM and resulted in an improvement in process indicators.

Process indicators related with access to lab tests and to ophthalmologists were very low at baseline but increased at 12 months and maintained at 24 months. The improvement of these indicators required reallocation of resources, and organizational changes within the healthcare centers. In our cohort, retinopathy was the most frequent microvascular complication at the baseline visit so although the proportion of participants who visited an ophthalmologist showed a statistically significant increase at 12 and 24 months compared to baseline, access to specialist care is still low.

The DP was effective considering the simultaneous improvements in the process indicators related to laboratory exams (Hb1Ac, cholesterol, and creatinine) which may imply that the attending physicians had more information to plan the individual management of T2DM patients.

Access to antidiabetic treatment, was high at baseline and increase at follow-up, with metformin being the most indicated drug 726/845 (85.9%), showing high adherence to the recommendations of the T2DM clinical practice guidelines. A 2009 Ministry of Health report on the use of chronic medication.T2DM showed a high variability in the prescription of antidiabetic medications and glibenclamide was the first line treatment in primary care [35].

Although there was an increase in the proportion of persons with T2DM treated and undergoing HbA1c check-ups, it did not result on better metabolic control showing that other factors might affect the management of persons with T2DM [36–38]. As reported in other studies, lack of treatment intensification and non-adherence make it difficult to obtain optimal metabolic control results, increase the incidence of complications and mortality; and generates higher health costs [39–41].

The inclusion of a diabetes registry system permitted the identification of aspects of diabetes care that were in need of improvement, highlighted the potential contribution of electronic health data to chronic care and increased awareness among health authorities regarding the limitations the health care system posed for persons with T2DM in terms of access to lab services and specialty care.

In addition, algorithms developed for the clinical support system generated alerts mainly focused on improving key processes of care. In this sense, results showed an improvement in process indicators at 12 and 24 months but not on glycemic control. There is evidence that clinical decision support systems for healthcare professionals can improve adherence to CPG, screening, referrals, efficiency and decrease medical errors [42, 43].

Unlike other studies in developing countries which demonstrated that SMS messaging had a positive impact on HbA1c, our study showed a marginal effect on glycemic control at 24 months [44, 45]. An overview of 15 systematic reviews concluded that mHealth interventions improved HbA1c in the short term in persons with T2DM.[15]. However, compared to our study these were of short duration. Similar results were reported by Van Olmen et al. in a randomized controlled trial conducted in three different LMIC where a text messaging intervention was implemented with a two year follow up period in existing diabetes programs [46]. A 3-arm RCT (*n* = 1372) in South Africa concluded that support through SMS could improve medication intake and had a small positive impact on blood pressure control compared to usual care in a general outpatient population of adults with hypertension [47].

Digital health interventions have become attractive to address the limitations of health systems in LMIC, mainly due to its accessibility, low cost, widespread use and applicability to a wide variety of health behaviors and conditions. Also, mobile phones are recognized as a viable platform to improve the provision of health services in low-income communities and for diabetes education. The integration of a digital health component in a public DP was innovative and well accepted among persons with T2DM who attend primary care clinics and hospitals [25]. Among health providers it was adopted by almost half of those initially trained.

Digital Health has recently gained attention in the context of the COVID-19 pandemic, as it enabled the optimization of service delivery platforms and environments. Technology is playing an important role in redesigning management strategies for chronic diseases during pandemic and lockdown [48, 49].

## Limitations

In our study, potential confounding variables might have been unmeasured and not controlled. This can only be properly controlled by the randomization process. Although pre-post interventions studies might be a threat to internal validity they are common in the medical informatics literature [50].

As for the implementation of the intervention, we detected problems related with access to lab services and special care (ophthalmologist visits) for patients attending the primary care level.

Access to the internet network as a condition for being selected as participating sites could limit the external validity of the findings, it is possible that the results cannot be generalized to other parts of the province or other environments. The success of any technology based intervention depends on how well it fits the needs of the users and the environment. In fact, some had technical infrastructure problems such as the lack of uninterrupted internet connectivity.

## Conclusion

In summary, the DP was innovative in Argentina, by integrating digital health, provider training and diabetes education in the public primary care level. An important component of the implementation of the program was the active identification of persons with T2DM and their inclusion into the Diabetes registry [12, 51, 52].

Although the study showed significant improvements in quality indicators related with the processes of diabetes care and in intermediate outcomes related with blood pressure control, we need to develop and implement new strategies to intensify treatment in a timely manner and coordinate an adequate follow-up.

## Data Availability

The datasets generated and/or analysed during the current study are not publicly available due institutional policy but are available from the corresponding author on reasonable request.
